# Septo-optic dysplasia and gastroschisis: trends in birth prevalence and association with maternal age

**DOI:** 10.1007/s00431-025-06118-4

**Published:** 2025-04-14

**Authors:** David J. Cullingford, Mary B. Abraham, Aris Siafarikas, A Marie Blackmore, Jenny Downs, Peter Jacoby, Gareth Baynam, Catherine S. Y. Choong

**Affiliations:** 1 Department of Endocrinology and Diabetes, Perth Children’s Hospital, 15 Hospital Avenue, Nedlands, Perth, WA 6009 Australia; 2 The Centre for Child Health Research, the Kids Research Institute Australia, University of Western Australia, Perth, WA Australia; 3 Faculty of Health & Medical Sciences, University of Western Australia, Perth, WA Australia; 4 The Centre for Child Health Research, University of Western Australia, Perth, WA Australia; 5 Institute of Health Research, University of Notre Dame, Fremantle, WA Australia; 6 School of Medicine & Health Sciences, Edith Cowan University, Mount Lawley, WA Australia; 7 Curtin School of Allied Health, Curtin University, Perth, WA Australia; 8 WA Department of Health, Genetic Services of Western Australia, Subiaco, WA 6008 Australia; 9 Western Australian Register of Developmental Anomalies, King Edward Memorial Hospital, PO Box 134, Subiaco, WA 6904 Australia

**Keywords:** Septo-optic dysplasia, Gastroschisis, Birth prevalence, Maternal age, Congenital

## Abstract

This study aims to describe the risk factors and trends in birth prevalence of septo-optic dysplasia (SOD) and gastroschisis between 1980 and 2023. This descriptive, population-based study of SOD and gastroschisis used Western Australian Register of Developmental Anomalies data from 1980 to 2023. Birth prevalence was calculated using Midwives Notification System data for all births after 20 weeks gestation. Relative risk (RR) for SOD and gastroschisis showed risk by maternal age. SOD and gastroschisis occurred in 99 and 391 cases respectively (no dual diagnoses), with a birth prevalence of 1.41 and 3.71 per 10,000 respectively (from 2010 to 2019), and have been stable since 2010. For the study period, younger maternal age (under 25 years old) increased the risk of SOD and gastroschisis (RR, 3.28 [95% confidence interval (CI) 2.13–5.00]; RR, 4.50 [95% CI 3.68–5.49] respectively). From 2010 to 2023, median maternal age increased for SOD and gastroschisis by 4.8 years (*p* = 0.044) to 27.0 and by 2.8 years (*p* < 0.001) to 25.5, respectively. Mothers living in areas of greater socioeconomic disadvantage had higher risk (RR, 1.81 [95% CI 1.14–2.88] in SOD and RR, 1.41 [95% CI 1.06–1.86] in gastroschisis), independent of maternal age. Premature birth was more common in children with SOD and gastroschisis. *Conclusion*: While younger mothers are more likely to have a child with either SOD or gastroschisis, maternal age at birth is higher than reported in the literature and is rising. This is probably attributable to the rising age of mothers at delivery, and further research into risk factors beyond maternal age is recommended.

**Table:** 

**What is Known:** *• Septo-optic dysplasia and gastroschisis are both rare diseases, known to occur more commonly in younger mothers.* *• Maternal age at birth is increasing in high income countries.* **What is New:** *• Despite mean maternal age rising in both conditions, in-line with rising population maternal age, birth prevalence is stable.* *• Mothers living in areas of greater socioeconomic disadvantage had a higher relative risk of SOD and gastroschisis.*

**What is Known:**

*• Septo-optic dysplasia and gastroschisis are both rare diseases, known to occur more commonly in younger mothers.*

*• Maternal age at birth is increasing in high income countries.*

**What is New:**

*• Despite mean maternal age rising in both conditions, in-line with rising population maternal age, birth prevalence is stable.*

*•  Mothers living in areas of greater socioeconomic disadvantage had a higher relative risk of SOD and gastroschisis.*

Septo-optic dysplasia (SOD) is defined by the presence of two or more of the following findings: optic nerve hypoplasia (ONH), hypopituitarism and midline defects (partial or complete agenesis of the corpus callosum or septum pellucidum). In the first trimester, the optic chiasm is immediately anterior to the developing pituitary; thus, both structures can be affected by an insult at this location [1]. SOD is the most common congenital cause of combined pituitary hormone deficiency [2], with birth prevalence internationally estimated at around 1 in 10,000 and increasing [3–5].

Aetiology for SOD is less understood, although genetics and antenatal environment both play parts. Several genetic causes have been identified, but these make up less than 10% of cases [6]. It has been observed consistently that children with SOD are frequently born to young, primiparous mothers, (median age 21 versus 29 years in the general population; 71% primiparous versus 43% in the general population, in a study from the UK [7]. These mothers typically live in areas with high rates of unemployment and social deprivation [3, 5]. Antenatal exposure to smoking, alcohol or recreational drug use have been proposed as risk factors, with some evidence to support potential mechanisms [8]; however, there is a lack of evidence to support causality [9].

Gastroschisis is a disorder of embryogenesis in the first trimester where the bowel protrudes through a periumbilical abdominal defect. Like SOD, it is more prevalent in children of young mothers, and birth prevalence is reported to be increasing [10]. Smoking, alcohol and recreational drug use are other known risk factors, while the impact of maternal socioeconomic status is unclear [11–14]. It is frequently detected on antenatal ultrasound, and has higher rates of intrauterine growth restriction, spontaneous premature labour, as well as elective induction of preterm labour compared to the general population [15, 16].

Maternal age in high-income countries is rising [17, 18], and little is known of the effect of this on birth prevalence of conditions seen more commonly in younger mothers. The impact of socioeconomic disadvantage on risk of SOD and gastroschisis also requires further investigation. Population-based datasets were used to investigate these questions because (a) they allowed linkage with maternal and socioeconomic data; (b) they provided birth-related data back to 1980, allowing analysis of trends over time; and (c) they maximized sample size for analyses of two conditions, both of which are rare.as b SOD and gastroschisis were examined together due to their shared risk factors, with this study aiming to examine their risk factors and trends in birth prevalence in Western Australia. Additionally, there are case reports of comorbid SOD and gastroschisis [19, 20], with one database study assessing the association previously [16]; thus, it was appropriate to report findings in parallel and assess for cases of dual diagnosis.

## Methods

This was a population-based retrospective cohort study of all children in Western Australia diagnosed with SOD or gastroschisis from 1980 to 2023. This includes those with prenatal diagnosis, stillbirths and termination of pregnancy (TOP) that were confirmed at birth, diagnosed postnatally or on postmortem.

### Data sources

Congenital anomaly data were extracted from the Western Australian Register of Developmental Anomalies (WARDA). WARDA, initiated in 1980, provides active surveillance of congenital anomalies for all births in Western Australia (WA), with a case ascertainment period from the in-utero period until 6 years of age. Diagnoses made after six can be recorded in remarks if the case was previously registered for another diagnosis before the sixth-year age cut-off, while cases diagnosed after cut-off age are recorded separately to avoid duplicate notification but not registered on the database. WARDA’s high quality data and findings are reported in > 325 peer-reviewed publications [21]. Aggregate data were also extracted from the WA Midwives Notification System (MNS), a statutory collection of newborn and maternal data recorded at the time of birth, also initiated in 1980.

### Variables

Extracted data including the child’s year of birth, sex, birth outcome (livebirth, stillbirth, or TOP), birth gestation and birth weight, as well as pregnancy plurality, maternal and paternal age and birth postcode were extracted from WARDA for the study cohort and the MNS for the general population. Age at diagnosis were extracted from WARDA. Parental and child date of birth were used to calculate parental age at delivery, classified in 5-year age group blocks. Postcode information was used to classify as either major city or rural/regional/remote, and socioeconomic status via the Australian Bureau of Statistics (ABS) Socio-Economic Indexes For Areas (SEIFA) Index of Relative Socio-Economic Disadvantage (IRSD) [22]. The IRSD is based on 15 measures of disadvantage (e.g. low income, joblessness, limited educational attainment) [22]. Age at diagnosis was classified as prenatal, neonatal (0–28 days); infancy (29–365 days); early childhood (1 to < 3 years); childhood (3–6 years); and late childhood (> 6 years). Maternal age was stratified in two ways: 5-year age group blocks, and as younger (defined as < 25 years of age) and older (defined as ≥ 25 years of age). The former to describe individual age group prevalence, as seen in other studies [4], the latter to allow cross-tabulation with social disadvantage (as the 5-year age bands would have produced cell sizes that were too small).

### Statistical analysis

Number and frequency (%) are used to describe demographic features with median and interquartile range utilised for continuous outcome measures. The Mann–Whitney *U* test is used to compare groups on continuous variables within a cohort (e.g. median maternal age for those born before and after 2010), Wilcoxon signed-rank test is used to compare continuous variables to population data, relative risk (RR) with 95% confidence intervals (CI) are used to reflect difference in risk between cohorts and test of proportions test is used to determine differences in prevalence rates. Adjusted relative risks using multivariate log binomial regression to compare maternal age categories (under and over 25) and IRSD quintiles was also employed. Calculations were performed using Stata version 18.0 (StatCorp, College Station, TX, USA).

Comparison of data between before and after 2010 were performed as case identification prior to 2000 in SOD was notably lower than after 2000, and data for 2018–2023 may be incomplete due to undetected cases. Overall case numbers are relatively low for SOD and use of at least a decade of data was felt appropriate.

For cases where characteristics data were missing, pairwise deletion was used. Terminations of pregnancy for foetal anomaly (TOPFA) prior to 20 weeks were excluded for the purpose of birth prevalence, gestational age and birth weight but were included for calculation of mean maternal age and socioeconomic status. For individuals whose SOD was not registered with WARDA until after age 6, clinical characteristics were not available, and so these individuals were included only in the calculations of total prevalence, year of birth and timing of diagnosis. Birth prevalence was defined as the total number of affected cases (including live and still births and TOPFA, divided by the total number of births (live and stillbirths)) in the population and expressed as the number per 10,000 as recommended by the World Health Organisation (WHO) [23]. For cases where TOPFA occurred, these cases were excluded from analysis of gestational age (as this was based on timing of detection, rather than natural delivery), and birthweight as this was not recorded. Data for WARDA cases were compared to aggregate population data from MNS. The MNS began collecting IRSD data for the Western Australian maternal cohort from 2006, and therefore comparisons of maternal risk by IRSD were only made for births from 2006 onwards.

Of note, the first magnetic resonance imaging (MRI) machine was installed at the paediatric tertiary centre in August 2003. Detection of septum pellucidum and corpus callosum abnormalities are typically required to make a diagnosis of SOD and require MRI or computer tomography (CT) imaging to diagnose postnatally. Therefore, cases born prior to 2003 may have had a diagnostic delay or remained undetected.

Ethics approval was obtained from the WA Department of Health Human Research Ethics Committee (RGS6499).

## Results

There were 1,213,092 births in WA across the study period, with 33.6% born to mothers under 25 years of age in 1980–1989 and 15.1% since 2010. There were 99 cases of SOD and 391 cases of gastroschisis (including 26 terminations) reported to WARDA with a median maternal age of 25.4 and 23.9 years, respectively (Table 1).

**Table 1:** Characteristics of the children

	Septo-optic dysplasia (*n* = 99)	Gastroschisis (*n* = 391)	Western Australian (*n* = 1,213,092)
Sex	34 F: 53 M (*n* = 87)	172 F: 211 M (*n* = 383)	622,871 F: 590,120 M (*n* = 1,212,991)
Birth prevalence			Not applicable
Total	0.81/10,000 (*n* = 99)	3.22/10,000(*n* = 391)	
2010–2019	1.41/10,000 (*n* = 47)	3.71/10,000 (*n* = 124)	
Maternal age (years)^a^	(*n* = 84)	(*n* = 389)	(*n* = 1,213,092)
Total	25.4 (20.6–30.4)*	23.9 (20.6–28.4)*	29 (25–33)
1980–2009	22.2 (19.6–28.5)*	22.7 (20.1–27.0)*	28 (24–32)
2010–2023	27.0 (22.7–31.8)*	25.5 (21.8–29.7)*	31 (27–34)
Difference	4.8 years (*p* = 0.044)	2.8 years (*p* < 0.001)	3 years
Paternal age (years)^a^	29.6 (23.7–33.9) (*n* = 53)	25.4 (22–32.8) (*n* = 16)	Not available
IRSD quintiles (since 2006)	(*n* = 73)	(*n* = 218)	(*n* = 581,476)
1 (most disadvantaged)	20.5%	18.3%	15.1%
2	30.1%	27.5%	17.2%
3	20.5%	22.9%	20.3%
4	15.1%	16.5%	26.2%
5 (least disadvantaged)	13.7%	14.7%	21.2%
Metropolitan area vs Other	64/23 (74.0% metropolitan)	285/105 (73.0% metropolitan)	74.5% metropolitan
Gestational age (weeks)^a^	39 (37.75–40) (*n* = 72)	36 (35–37)* (*n* = 355)	39 (38–40) (*n* = 1,213,092)
≥ 37 weeks	77.8%	41.0%	92.7%
32–36 weeks	12.5%	55.1%	6.2%
28–31 weeks	5.6%	2.5%	0.7%
< 28 weeks	4.2%	1.4%	0.4%
Birth weight^a^ (kilograms) > 37 weeks gestation	3.12 (2.64–3.63) (*n* = 68) 3.32 (3.03–3.70) (*n* = 52)	2.49 (2.11–2.76) (*n* = 354) 2.68 (2.45–2.99)* (*n* = 146)	Not available 3.42 (3.12–3.73)
Birth outcome	(*n* = 87)	(*n* = 391)	(*n* = 1,213,092)
Live birth	100%	88.5%	99.3%
Stillbirth	0%	4.9%	0.7%
TOPFA	0%	6.6%	
Time to diagnosis	(*n* = 99)	(*n* = 391)	Not applicable
Prenatal	3.0%	89.0%	
Neonatal	26.3%	11.0%	
Infancy	30.3%	0%	
1–5 years	27.3%	0%	
Over 6 years	13.1%	0%	

^a^Median and interquartile range

^*^Statistically significant difference compared to WA population data *p* < 0.05

Out of the 99 SOD cases, 12 were notified to WARDA after the age of 6 years due to late diagnosis and hence could not be registered. Therefore, clinical characteristics were available to be assessed for the 87 registered cases, with prevalence based on 99 cases. There were no cases with a dual diagnosis of SOD and gastroschisis.

### Birth prevalence of SOD and gastroschisis over study period

**Fig. 1:**
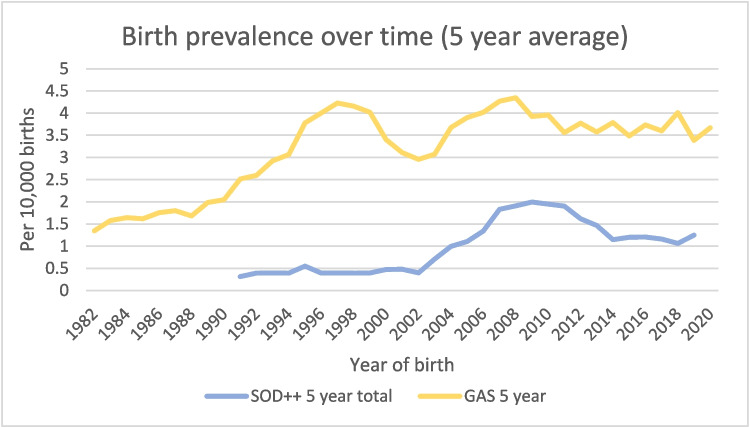
Birth prevalence over time. The 5-year period birth prevalence is calculated to represent trends overtime. Prevalence is recorded at the middle year of the 5-year period (e.g. prevalence for 2000–2004 reported as 2002). SOD data between 1980 and 1991 censored due to either no cases, or case numbers ≤ 3 per 5 years

For SOD, birth prevalence increased significantly from 1990–1999 to 2000–2009 (0.40 to 1.19/10000 births), with no significant increase between 2000–2009 and 2010–2019 (Table 2). For gastroschisis, birth prevalence significantly increased from 1980–1989 to 1990–1999 (1.60 to 3.46/10000 births), with no significant difference between subsequent decades. Figure 1 represents the 5-year average of birth prevalence and rise in prevalence across the study period.

**Table 2:** Birth prevalence, risk factors and associations

	Septo-optic dysplasia			Gastroschisis		
	Under 25	Over 25	Total	Under 25	Over 25	Total
Birth prevalence (per 10,000 births)						
Total	1.52 (1.09–2.07)	0.46 (0.33–0.61)	0.82 (0.66–0.99)	8.09 (7.06–9.25)	1.80 (1.54–2.09)	3.20 (2.91–3.56)
1980–1989	[< 4 cases]	[< 4 cases]	[< 4 cases]	2.84 (1.78–4.29)	0.98 (0.55–1.61)	1.60 (1.13–2.21)
1990–1999	[< 4 cases]	[< 4 cases]	0.40 (0.19–0.73)	9.36 (7.15–12.05)	1.44 (0.95–2.10)	3.46 (2.77–4.27)
2000–2009	2.60 (1.45–4.28)	0.47 (0.23–0.87)	1.19 (0.82–1.68)	10.21 (7.77–13.17)	1.90 (1.36–2.58)	3.69 (2.30–4.49)
2010–2019	3.30 (1.95–5.21)	0.97 (0.64–1.4)	1.41 (1.03–1.87)	11.54 (8.87–14.76)	2.18 (1.67–2.80)	3.71 (3.08–4.42)
Time-based relative risk						
2010–2019 vs 2000–2009	1.27 (0.64–2.52)	2.03 (0.98–4.2)	1.18 (0.75–1.85)	1.13 (0.79–1.61)	1.15 (0.77–1.71)	1.00 (0.77–1.31)
2010–2023 vs 1980–2009	**3.31 (1.79–6.11)**	**3.74 (1.93–7.26)**	**2.83 (1.82–4.39)**	**1.55 (1.18–2.06)**	**1.51 (1.11–2.03)**	1.20 (0.98–1.47)
Socioeconomic disadvantage relative risk						
1st or 2nd quintile (most disadvantaged) vs 3rd–5th quintile	1.35 (0.67–2.70)	**2.25 (1.22–4.15)**	**2.15 (1.36–3.41)** Adjusted for maternal age **1.81 (1.14–2.88)**	**1.56 (1.07–2.29)**	1.13 (0.74–1.72)	**1.77 (1.36–2.31)** Adjusted for maternal age **1.41 (1.06–1.86)**
4th or 5th quintile (least disadvantaged) vs 1st–3rd quintile	0.65 (0.29–1.44)	**0.46 (0.24–0.90)**	**0.45 (0.27–0.74)** Adjusted for maternal age **0.52 (0.31–0.87)**	**0.46 (0.290–0.75)**	0.73 (0.49–1.09)	**0.50 (0.38–0.67)** Adjusted for maternal age **0.60 (0.45–0.81**)

95% confidence interval are reported in brackets

Boldface indicates a statistically significant difference in relative risk between groups

### Birth prevalence of SOD and gastroschisis by maternal age

Children born to younger mothers had a RR of 3.29 (95% CI 2.13–5.00) compared to mothers over 25 years for SOD, while in gastroschisis, younger mothers had a RR of 4.50 (95% CI 3.68–5.49), with no significant change between decades. This association persists when adjusting for socioeconomic status as measured by IRSD (births from 2006 onwards only) with an RR of 3.64 (2.28–5.80) and 5.08 (3.85–6.70). Birth prevalence was highest in the < 20 age group (5.84 and 16.78/10,000 in SOD and gastroschisis respectively), with decreasing rates until age 30 in SOD and 35 in gastroschisis, where birth prevalence stabilised (Table 3).

**Table 3:** Birth prevalence (per 10,000 births) by maternal age group for SOD and gastroschisis (2000–2019)

	Maternal age group						
	< 20	20–24	25–29	30–34	35–39	< 25	≥ 25
SOD	5.84* (3.33–9.49)	2.10* (1.30–3.21)	0.90* (0.53–1.42)	0.57 (0.31–0.95)	0.54 (0.21–1.11)	2.94** (2.02–4.12)	0.75 (0.53–1.04)
Gastroschisis	16.78** (12.29–22.38)	8.99** (7.22–11.04)	3.64** (2.85–4.58)	1.66* (1.2–2.26)	0.84 (0.42–1.51)	10.66** (8.95–12.61)	2.12 (1.77–2.52)

^*^Statistically significant difference in prevalence from subsequent age group—*p* < 0.05

^**^Statistically significant difference in prevalence from subsequent age group—*p* < 0.001

### Birth prevalence of SOD and gastroschisis by study decades

There was no rise in RR when comparing 2000–2009 and 2010–2019 for younger or older mothers with SOD or gastroschisis (Table 2). However, comparing before and after 2010 (1980–2009 to 2010–2023), the age-associated RR rose for SOD and gastroschisis (in younger and older mothers) with change similar in both groups.

For SOD, 19/31 (61.2%) were born to mothers under 25 prior to 2010 to 22/54 (41.7%) since the start of 2010. The change in median maternal age over this period was an increase of 4.8 years, *p* = 0.044 (Table 1). Approximately two-thirds (*n* = 142/225, 63.5%) of children with gastroschisis were born to mothers under 25 prior to 2010, compared to 77/166 (46.4%) since the start of 2010, and median maternal age increased 2.8 years (*p* < 0.001), with a change of 3 years noted in the WA general population (see Table 1).

### Family residence

There was no difference in proportion of children to mothers from rural locations in either condition when compared to the general population. There were no associations between residence location and gestational age, birthweight or timing of diagnosis. Socioeconomic status, as reflected by IRSD, was associated with increased RR, with Table 2 demonstrating increased RR of SOD and gastroschisis in mothers in the two most disadvantaged quintiles, and a decreased RR in the two least disadvantaged quintiles. This pattern persisted when calculations were adjusted for maternal age (Table 2).

### Pregnancy and birth characteristics

Gastroschisis was associated with a lower median gestational age (36 vs 39 weeks gestation) as shown in Table 1, with greater RR of premature birth (< 37 weeks) compared to the general population in gastroschisis and SOD (Tables 1 and 4). This risk persisted when cases of prenatally diagnosed gastroschisis were excluded (RR 5.45 [95% CI 3.63–8.17]), although those with a postnatal diagnosis were more likely to reach term (RR 1.54 [95% CI 1.13–2.09]) with 21/35 delivered at term. The RR of prematurity below 32 weeks was also greater in SOD and gastroschisis (Table 4).

**Table 4:** Relative risk of maternal and neonatal features compared to the general population

	Septo-optic dysplasia	Gastroschisis
Maternal age < 25 years	**2.20 (1.77–2.74)**	**2.53 (2.32–2.76)**
Prematurity (weeks)		
< 37	**3.03 (1.96–4.66)**	**7.94 (7.26–8.70)**
32–367	**2.02 (1.10–3.72)**	**8.99 (8.17–9.89)**
< 32	**8.45 (4.18–17.10)**	**2.33 (1.22–4.44)**
Low birth weight at term (< 2500 g)	1.91 (0.49–7.43)	**15.98 (12.53–20.38)**
Stillbirths	No cases	**7.77 (5.01–12.04)**

95% confidence interval are reported in brackets

Boldface indicates a statistically significant difference in relative risk from the general population

Stillbirths and terminations were not seen in the SOD cohort. For gastroschisis 4.9% of affected pregnancies resulted in stillbirth, significantly higher than the general population (Table 4). Of the 145 pregnancies persisting to term, five resulted in stillbirth with 8/187 pregnancies between 32- and 37-week gestation. 6.6% pregnancies affected by gastroschisis were terminated, with a higher median maternal age in terminated cases (26.1 years [22.3–30.6] vs 23.6 years [20.3–28.3], *p* = 0.017).

**Fig. 2:**
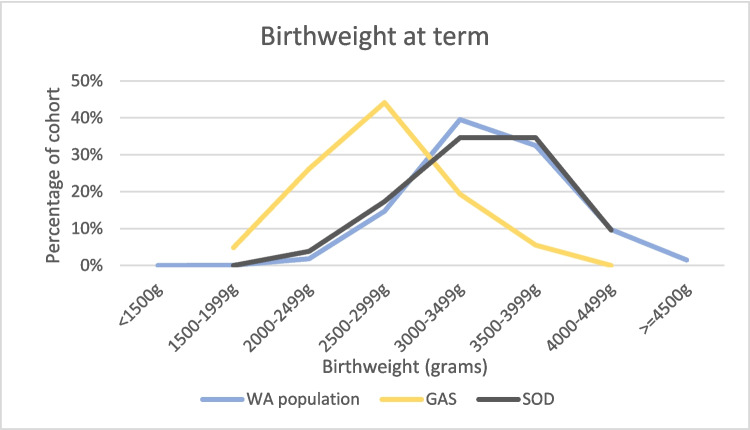
Birthweight of babies delivered at term, Western Australian (WA), gastroschisis (GAS) and SOD

Median birthweight at term was similar to the general population in SOD, but lower in gastroschisis (Table 1 and Fig. 2), with 45/145 being low birth weight (< 2500 g), and an increased RR of low birth weight (Table 4).

### Timing of diagnosis

Gastroschisis was diagnosed antenatally or in the neonatal period in 100%, and SOD in 29.3% of cases. 13.1% of cases of SOD were diagnosed after the age of 6 years, with 5/15 children born prior to the availability of MRI in 2003 and 8/84 cases born thereafter (RR 3.50 [95% CI 1.32–9.26]). There was no association between maternal age, IRSD or rural or metropolitan location regarding timing of diagnosis for SOD or gastroschisis.

## Discussion

These population-based data about the epidemiology of SOD and gastroschisis over time demonstrate that, while children of younger mothers have a higher birth prevalence of these conditions, they are now a minority of the patient cohort. While increasing maternal age across the Western Australian population may be responsible for part of this, birth prevalence in mothers in each age group is increasing, resulting in total birth prevalence being stable over the past 2 decades. Mothers living in areas of greater socioeconomic disadvantage were more likely to have an affected child, independent of maternal age. Both cohorts had higher risk of prematurity than the general population, with over half of gastroschisis cases being born premature. Low birth weight was more common in gastroschisis but not in SOD. There were no cases who had dual diagnosis, as seen in a recent study [16].

### Association with maternal age

The association between SOD and young maternal age is well established, with most studies reporting median ages between 20 and 21 [7, 24]. EUROCAT, a network of European congenital anomaly registries, report of children born between 2005 and2014 found that mothers aged 20–24 are more likely to have children with SOD than mothers aged 15–19 (9 versus 7 per 100,000 respectively) [4]. After 25 years of age, the birth prevalence becomes lower with each 5-year period. While the present study also demonstrates falling birth prevalence with increasing maternal age, the peak was found in the 15–19-year cohort, which is consistent with younger mean maternal ages reported in other studies [3, 5].

### Diagnosis/case ascertainment

Gastroschisis was diagnosed antenatally in 89% of cases, consistent with other studies [25], while in SOD, this was uncommon (3%), with cases detected across childhood. This is likely to be due to the presenting features, with gastroschisis typically being evident on antenatal ultrasound or seen at birth due to the visible external bowel, while SOD has a more variable diagnostic pathway. In SOD, while antenatal diagnosis based on absent septum pellucidum on ultrasound, or neonatal diagnosis of hypopituitarism or neurological sequelae of associated features occur, it is usually identified later due to vision, developmental or growth impairments in childhood. This variability may also result in incomplete case ascertainment for SOD, particularly in registers which only ascertain in infancy. MRI is typically required to make the diagnosis after infancy, and thus the impact of this becoming available in 2003 at our centre may have contributed to the distinct rise in case identification.

### Changing prevalence

Rates of SOD increased from 1990–1999 levels to 2000–2009 and were stable across the subsequent decade, with a similar pattern seen in gastroschisis, with the rise between 1980–1989 and 1990–1999. Birth prevalence of SOD is reported to be increasing [5], in gastroschisis change varies in other studies: some reporting increases [12, 13], while the EUROCAT register reports a decrease since 2010. Younger mothers and mothers over 25 years of age had a rise in birth prevalence when comparing before and after 2010, similar to what is reported in other studies [26]. Due to the reduction in rates of younger mothers however this rise in risk is not reflected in a rise in overall birth prevalence in the past two decades.

### Risk factors

The finding that birth prevalence increased across the study period despite (a) a 40% reduction in young mothers between the 1990 s and 2010–2023 and (b) similar rates of increase in RR for both younger mothers and mothers over 25 years of age supports the hypothesis that young maternal age is a marker of other underlying risk factors for SOD and gastroschisis. It suggests that while women are waiting until they are older to have children, these risk factors still purvey risk as they age. Mothers living in areas of socioeconomic disadvantage had a greater RR of both conditions, including when adjusted for maternal age. Socioeconomic disadvantage is known to be associated with SOD [3, 5], although the evidence in gastroschisis is more variable [14]. Lower pre-pregnancy BMI and poor first and second trimester weight gain have been seen in other studies [27, 28], which suggest nutrition may be an important driver in both conditions. Smoking and drug use have also been suggested as risk factors [14, 29]. In Western Australia, rates of maternal underweight status are similar over time at 2% (2.2% in 2012–2021 and below the national average 2.7% [17]), and maternal smoking decreased from 21.5 to 7% of pregnancies (2001–2021) [17, 30]. These findings should support a decrease in prevalence, rather than the rise seen in our cohort.

### Birth outcomes

Preterm birth is commonly seen in gastroschisis (22–90%) [31, 32] and SOD [5, 29], and the findings of the present study support these associations. Children with gastroschisis were still more likely to be preterm if diagnosis was made at birth, reflecting that gastroschisis itself purveys an increased risk of prematurity and not only secondary to elective pre-term delivery to minimise risk of stillbirth. Gastroschisis is also associated with low birth weight and elevated risk of stillbirth and mothers who elect for TOPFA are typically older, with the findings in the present cohort similar to findings from other studies [13, 25, 32].

### Limitations

As SOD typically requires neuroimaging for diagnosis, the reported birth prevalence prior to the availability of MRI in 2003 is likely to be influenced by under ascertainment. Even after the time of introduction of MRI, some cases are not diagnosed until after 6 years of age and would therefore not be registered with WARDA. Cases of SOD diagnosed after age 6 (12% of the sample) could not be included in most of the analyses due to an absence of linked data; there are insufficient data about these children to know whether or how they differed from those diagnosed before age 6. We were unable to assess the impact of smoking, alcohol and drug use, maternal parity and BMI as these variables were not available in the dataset. A more comprehensive examination of risk factors is recommended.

## Conclusion

In summary, birth prevalence of septo-optic dysplasia and gastroschisis is higher in younger mothers and decreases as women age in the present cohort. Birth prevalence increased across all age groups in previous decades for both septo-optic dysplasia and gastroschisis in Western Australia. Notably, birth prevalence has remained stable since 2010, probably due to increasing maternal age at birth, into age groups where risk is lower. Mothers of children with septo-optic dysplasia and gastroschisis are more frequently from areas of greater social disadvantage and babies are frequently born preterm, with low birthweight and stillbirths more common in gastroschisis. We propose that while women are waiting until they are older to have children, other underlying risk factors persist, and further studies into risk factors beyond, or that mediate the effect of, maternal age are needed.

## Data Availability

No datasets were generated or analysed during the current study.

## Abbreviations

ABS: Australian Bureau of Statistics
CI: Confidence Interval
CT: Computer tomography
IRSD: Index of Relative Socio-Economic Disadvantage
MNS: Midwives Notification System
MRI: Magnetic Resonance Imaging
ONH: Optic nerve hypoplasia
RR: Relative risk
SOD: Septo-optic dysplasia
SEIFA: Socio-Economic Indexes For Areas
TOPFA: Terminations Of Pregnancy for Fetal Anomaly
WA: Western Australia
WARDA: Western Australian Register of Developmental Anomalies
